# Travel-related *Neisseria meningitidis* Serogroup W135 Infection, France

**DOI:** 10.3201/eid1906.120515

**Published:** 2013-06

**Authors:** Muhamed-Kheir Taha, Adèle Kacou-N’Douba, Eva Hong, Ala Eddine Deghmane, Dario Giorgini, Sophia Lurette Okpo, Tatiana Kangah, Mireille Dosso

**Affiliations:** Institut Pasteur, Paris, France (M.-K. Taha, E. Hong, A.E. Deghmane, D. Giorgini);; Institut Pasteur, Abidjan, Côte d’Ivoire (A. Kacou-N’Douba, S.L. Okpo, T. Kangah, M. Dosso)

**Keywords:** Epidemic, meningitis, W135, Africa, bacteria, Neisseria meningitidis, serogroup, outbreak, travel, France, sub-Saharan Africa, serogroup W135

**To the Editor:** A multinational outbreak of infection with *Neisseria meningitidis* serogroup W135 belonging to the sequence type (ST) 11 clonal complex started in the year 2000 among pilgrims to Mecca, Saudi Arabia, and their contacts and continued in 2001 in countries of sub-Saharan Africa (primarily Burkina Faso) (*1*). Thereafter, infection caused by these isolates decreased (*2*), but quadrivalent meningococcal vaccine (against serogroups A, C, Y, and W135) was recommended for pilgrims and travelers to countries in the meningitis belt of Africa, which spans sub-Saharan Africa from Ethiopia to Senegal. After 2001, infections caused by serogroup A predominated in the meningitis belt, but isolates of serogroup X also emerged (*3*); isolates of serogroup W135/ST11 increased again in Niger in 2010 (*4*). 

During January 1–March 11, 2012, >4,000 suspected cases of meningococcal disease caused mainly by serogroup W135 were reported in countries of the African meningitis belt, including Benin, Burkina Faso, Mali, and Côte d’Ivoire (*5*). We present extensive bacteriologic and molecular characterization of *N. meningitidis* W135 isolates from 6 patients with meningococcal disease reported in France since January 2012; we also present typing data from 8 cases of meningitis in Côte d’Ivoire. None of the patients had received meningococcal vaccine. 

The cases in France were neither epidemiologically nor geographically linked; 4 were in residents of the Paris region. All cases were linked to recent travel to sub-Saharan Africa by the patient or patient contacts; 4 patients reported recent travel to Benin, Senegal, or Mali (Table). The 2 other cases were in a 4-month-old infant whose father had returned from Senegal 2 weeks before the onset of the disease and in a 5-year-old child who had several family members who visited Mali regularly, although no recent travel was documented. The delay between the return to France and the onset of the disease was <5 days except for 1 patient (17 days). 

**Table:** Characteristics of serogroup W135 *Neisseria meningitidis* isolates from patients in France and Côte d'Ivoire, 2012

Patient no.	Location†	Date of illness onset	Travel history	Date of return from travel	Patient age/sex	Test site	*N. meningitidis* isolate test results						
							Sero	ST	CC	PorA VR1	PorA VR2	FetA	*penA*
1	France/ Paris region	Jan 14	Benin	2011 Dec 28	1 y/M	CSF	2a	11	11	5	2	F1-1	1
2	France/ Loire	Feb 15	Senegal	Feb 12	62 y/F	Blood	2a	11	11	5	2	F1-1	1
3	France/ Rhône-Alpes	Feb 19	Senegal	Feb 19	53 y/F	Blood	2a	11	11	5	2	F1-1	1
4	France/ Paris region	Feb 27	Mali	Feb 23	4 y/M	AF	2a	11	11	5	2	F1-1	1
5	France/ Paris region	Mar 3	Mali (family)	UNK	5 y/F	CSF	2a	11	11	5	2	F1-1	1
6	France/ Paris region	Mar 6	Senegal (father)	Feb 26	4 mo/F	CSF	2a	11	11	5	2	F1-1	1
7	Côte d'Ivoire/ Kouto	Feb 3	UNK	NA	12 y/M	CSF	2a	11	11	5	2	F1-1	1
8	Côte d'Ivoire/ Korhogo	Feb 3	UNK	NA	18 y/M	CSF	2a	11	11	5	2	F1-1	1
9	Côte d'Ivoire/ Kouto	Feb 23	UNK	NA	7 y/F	CSF	2a	11	11	5	2	F1-1	1
10	Côte d'Ivoire/ Kouto	Feb 24	UNK	NA	65 y/F	CSF	2a	11	11	5	2	F1-1	1
11	Côte d'Ivoire/ Kouto	Feb 24	UNK	NA	1 y/F	CSF	2a	11	11	5	2	F1-1	1
12	Côte d'Ivoire/ Tengrela	Feb 24	UNK	NA	19 y/F	CSF	2a	11	11	5	2	F1-1	1
13	Côte d'Ivoire/ Tengrela	Feb 24	UNK	NA	3 y/M	CSF	2a	11	11	5	2	F1-1	1
14	Côte d'Ivoire/ Tengrela	Feb 24	UNK	NA	5 y/M	CSF	2a	11	11	5	2	F1-1	1

*All patients had meningitis except patients 2 and 3, who had bronchopneumonia and septicemia, and patient 4, who had arthritis. Sero, serotype; ST, sequence type, CC, clonal complex; VR, variable region; UNK, unknown; CSF, cerebrospinal fluid; AF, articular fluid; NA, not applicable. †Country/region where case was reported.

*N. meningitidis* isolates were recovered from blood, cerebrospinal fluid, or articular fluid from all 6 patients in France (Table). Two patients had septicemia after bronchopneumonia, but no respiratory samples were available. One patient had arthritis that was also described in his sister, but no samples were available from the sister. Extrameningeal forms of illness caused by W135/ST11 isolates have been described (*6*). For the patients in Côte d’Ivoire, bacteria were isolated from cerebrospinal fluid during weeks 5–8 in 2012; the patients lived in 3 districts of the country (Kouto, Korhogo, and Tengrela). Mean age was 20.9 years (range 0.33–62) for the patients in France and 16.25 years (range 1–65) for those in Côte d’Ivoire. 

Molecular typing was performed by multilocus sequence typing (MLST) using the PubMLST database (http://pubmlst.org/neisseria); typing included the 7 usual genes of MLST, PorA variable regions 1 and 2, and *penA* and *fetA* genes. Results were obtained by using cultured bacteria for all but 1 case in France. All isolates from France and Côte d’Ivoire shared the same tested markers. Eight other cases of infection with serogroup W135 were found in France during the same period, but the patients had no travel history, and all isolates showed different markers (M.-K. Taha, unpub. data).

Serogroup W135 strains are widely distributed worldwide; the emergence of these strains during the 2000s corresponded to a clonal expansion of 1 clone within the ST11 complex (*7*). The subsequent decline of W135/ST11 strains was associated with increased isolate diversification (*8*), which suggests a selective restriction of the dominant circulating strain (*2*). In France, the W135/ST11 strain was rare after 2005; no cases were culture confirmed in 2010, and the 2 cases that were confirmed in 2011 showed the FetA2-19 marker. However, isolates from Africa during 2000–2011 frequently showed the FetA1 marker; in sub-Saharan Africa, the decline of W135/ST11 isolates was also associated with isolates showing diversified the FetA marker (M.-K. Taha, unpub. data). The reemergence in 2012 of W135/ST11 strains that had FetA1-1 markers suggests an antigenic shift that may have involved membrane proteins other than FetA or other surface structures, such as the lipooligosasccharide. Such antigenic shifts were associated with increased incidence of serogroup C and serogroup Y meningococcal disease in the United States (*9*). Antigenic shift could be a marker of changes in virulence and transmission of meningococcal isolates. Extensive molecular typing of meningococcal isolates is more likely to detect antigenic shifts and escape variants that may undergo clonal expansion and therefore should be employed in outbreak investigations. Enhanced surveillance was setup in France to identify imported W135 cases.

Our findings indicate that travelers to the meningitis belt of sub-Saharan Africa may be at risk for infection with *N. meningitidis* of serogroup W135. A vaccination campaign using the meningococcal A conjugate vaccine is ongoing in this region (*10*), but a conjugate bivalent vaccine that includes W135 should also be considered. Vaccination of travelers to this region with quadrivalent meningococcal vaccine should be recommended. 
